# Crystal structure and C—H⋯F hydrogen bonding in the fluorinated bis-benzoxazine: 3,3′-(ethane-1,2-di­yl)bis­(6-fluoro-3,4-di­hydro-2*H*-1,3-benzoxazine)

**DOI:** 10.1107/S2056989016015243

**Published:** 2016-09-30

**Authors:** Augusto Rivera, Jicli José Rojas, Jaime Ríos-Motta, Michael Bolte

**Affiliations:** a Universidad Nacional de Colombia, Sede Bogotá, Facultad de Ciencias, Departamento de Química, Cra 30 No. 45-03, Bogotá, Código Postal 111321, Colombia; b Institut für Anorganische Chemie, J. W. Goethe-Universität Frankfurt, Max-von Laue-Strasse, 7, 60438 Frankfurt/Main, Germany

**Keywords:** crystal structure, benzoxazine, non-conventional hydrogen bonds

## Abstract

The title fluorinated bis­benzoxazine, C_18_H_18_F_2_N_2_O_2_, crystallizes with one half-mol­ecule in the asymmetric unit, which is completed by inversion symmetry. The fused oxazine ring adopts an approximately half-chair conformation. The two benzoxazine rings are oriented *anti* to one another around the central C—C bond. The dominant inter­molecular inter­action in the crystal structure is a C—H⋯F hydrogen bond between the F atoms and the axial H atoms of the OCH_2_N methyl­ene group in the oxazine rings of neighbouring mol­ecules. C—H⋯π contacts further stabilize the crystal packing.

## Abstract 2

The packing of the title benzoxazine derivative features C—H⋯F hydrogen bonds, which form a sheet structure further linked *via* weak C—H⋯π hydrogen bonds.

## Chemical context   

Even though benzoxazines have been known for more than 60 years, a cursory look at the literature cited in relation to the polybenzoxazines in recent years reveals increasing inter­est in polybenzoxazine chemistry (Demir *et al.*, 2013 ▸). Mannich condensation of a phenol and a primary amine with formaldehyde is perhaps the best synthetic route widely employed for the preparation of a variety of benzoxazine monomers. Mono-functional benzoxazines with one oxazine ring yield linear polymers, while bi- and polyfunctional benzoxazines produce cross-linked polymers. As a result, many kinds of benzoxazine monomers, including both mono-benzoxazines and bis-benzoxazines, have been synthesized. For composite applications, bifunctional benzoxazines are important as they produce fillers with good adhesion properties that in turn give high modulus composite materials (Santhosh-Kumar & Reghunadhan-Nair, 2014 ▸).
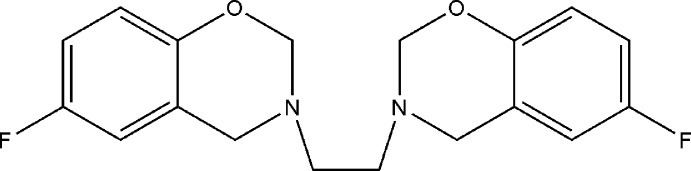


Much work in our group has been directed at the synthesis of a wide variety of bis-benzoxazines from ethyl­endi­amine, formaldehyde and phenols in the molar ratio of 1:4:2 using a conventional method and solvent-free conditions (Rivera *et al.*, 1989 ▸). Recently, we have also investigated the crystal structures of several bis-benzoxazines namely 3,3′-(ethane-1,2-di­yl)bis­(6-substituted-3,4-di­hydro-2*H*-1,3-benzoxazine) derivatives (Rivera *et al.*, 2010 ▸, 2011 ▸, 2012*a* ▸,*b* ▸). These were prepared to determine whether replacement of the substit­uents at the *para* position of the phenol affects the mol­ecular conformation and possible supra­molecular aggregation. In this context, the title compound is a model for studying non-conventional mol­ecular inter­actions where the halogen atom may act as a hydrogen-bond acceptor. Although debate has surrounded the role of fluorine as a hydrogen-bond (HB) acceptor, the presence of such weak mol­ecular inter­actions in the solid state has been the subject of both theoretical and spectroscopic studies (Dalvit & Vulpetti, 2016 ▸). However, to the best of our knowledge, there are few examples of X-ray studies. On the other hand, polymers containing fluorinated aromatic systems often exhibit exceptional thermal stability and show good water-repellent properties (Su & Chang, 2003 ▸). Therefore we report herein the crystal structure of 3,3′-(ethane-1,2-di­yl)bis­(6-fluoro-3,4-di­hydro-2*H*-1,3-benzoxazine) (I), which is a very good candidate as a monomer for the investigation of the polymerization of fluorine-containing bis-benzoxazine monomers.

## Structural commentary   

**Figure 1:**
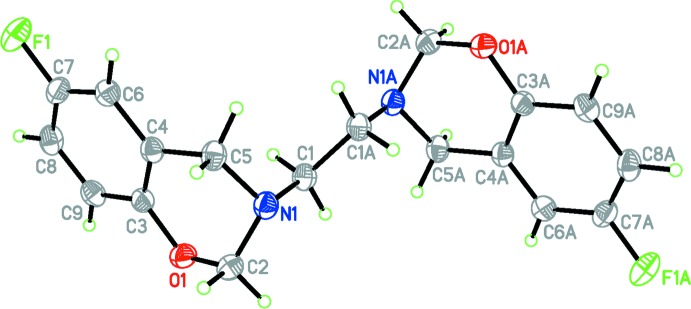
The mol­ecular structure of the title compound. Displacement ellipsoids are drawn at the 50% probability level. Atoms labelled with the suffix A are generated using the symmetry operator (−*x* + 1, −*y* + 1, −*z* + 1).

The mol­ecular structure of the title compound is illustrated in Fig. 1 ▸. The asymmetric unit contains one-half of the formula unit; a centre of inversion located at the mid-point of the central C1—C1^i^ bond generates the other half of the bis-benzoxazine compound [symmetry code: (i) 1 − *x*, 1 − *y*, 1 − *z*]. Bond lengths in the benzoxazine moiety in (I) are within normal ranges and are comparable to those found in related structures (Rivera *et al.*, 2012*a* ▸,*b* ▸, 2011 ▸; Chen & Wu, 2007 ▸).

The fused six-membered heterocyclic rings exist in an approximately half-chair conformation, characterized by a puckering amplitude *Q* = 0.4913 (15) Å, and θ = 52.03 (17)° and φ = 98.3 (2)°, with C2 and N1 displaced from the mean plane by −0.299 (2) and 0.331 (1) Å, respectively. The C1—C1*A* bond is in an axial position with a C5—N1—C1—C1*A* torsion angle of 75.45 (18)°. The two benzoxazine rings are oriented *anti* to one another about the central C1—C1*A* bond, with an N1—C1—C1*A*—N1*A* torsion angle of 180.0 (2)°.

## Supra­molecular features   

**Figure 2:**
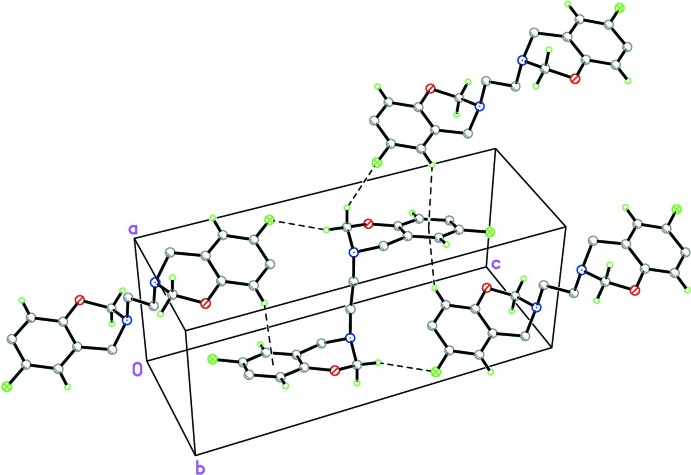
Packing diagram for title compound, viewed along the *b* axis. C—H⋯F and C—F⋯π contacts are drawn as dashed lines.

The packing of title compound is dominated by C2—H2*A*⋯F1 hydrogen bonds (Table 1 ▸), that connect the mol­ecules into a sheet structure, Fig. 2 ▸. Symmetry dictates that both F atoms are involved in these hydrogen bonds. The crystal structure also features two weak C—H⋯π inter­actions (Table 1 ▸), as indicated in *PLATON* (Spek, 2009 ▸), with C—H⋯*Cg* distances of 3.527 (2) and 3.577 (2) Å and with C—H⋯*Cg* angles of 126 and 129°, respectively.

## Database survey   

A database search yielded four comparable structures, 3,3′-(ethane-1,2-diyl)bis(6-methyl-3,4-di­hydro-2*H*-1,3-benzoxazine) (AXAKAM; Rivera *et al.*, 2011 ▸), 3,3′-ethyl­enebis(3,4-di­hydro-6-chloro-2*H*-1,3-benzoxazine) (NUQKAM; Rivera *et al.*, 2010 ▸), 3,3′-(ethane-1,2-di­yl)-bis­(6-meth­oxy-3,4-di­hydro-2*H*-1,3-benzoxazine) monohydrate (QEDDOU; Rivera *et al.*, 2012*b* ▸), and 3,3′-(ethane-1,2-diyl)bis(3,4-di­hydro-2*H*-1,3-benz­oxazine) (SAGPUN; Rivera *et al.*, 2012*a* ▸).

## Synthesis and crystallization   

The title compound was synthesized according to the literature procedure (Rivera *et al.*,1989 ▸), and single crystals were obtained by slow evaporation from an ethyl acetate/benzene 1:3 solvent mixture at room temperature.

## Refinement details   

Crystal data, data collection and structure refinement details are summarized in Table 2 ▸. All H atoms were located in difference electron-density maps. C-bound H atoms were fixed geometrically (C—H = 0.95 or 0.99 Å) and refined using a riding-model approximation, with *U*_iso_(H) set to 1.2*U*_eq_ of the parent atom.

## Supplementary Material

Crystal structure: contains datablock(s) I. DOI: 10.1107/S2056989016015243/sj5506sup1.cif

Structure factors: contains datablock(s) I. DOI: 10.1107/S2056989016015243/sj5506Isup2.hkl

Supporting information file. DOI: 10.1107/S2056989016015243/sj5506Isup3.cml

CCDC reference: 1507056

Additional supporting information: crystallographic information; 3D view; checkCIF report

## Figures and Tables

**Table 1:** Hydrogen-bond geometry (Å, °) *Cg*2 is the centroid of the C3/C4/C6/C7/C8/C9 ring

*D*—H⋯*A*	*D*—H	H⋯*A*	*D*⋯*A*	*D*—H⋯*A*
C2—H2*A*⋯F1^i^	0.99	2.44	3.300 (2)	145
C6—H6⋯*Cg*2^ii^	0.95	2.88	3.527 (2)	126
C9—H9⋯*Cg*2^iii^	0.95	2.90	3.577 (2)	129

Symmetry codes: (i) 
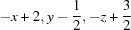
; (ii) 
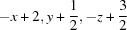
; (iii) 
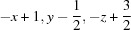
.

**Table 2:** Experimental details

Crystal data	
Chemical formula	C_18_H_18_F_2_N_2_O_2_
*M*_r_	332.34
Crystal system, space group	Monoclinic, *P*2_1_/*c*
Temperature (K)	173
*a*, *b*, *c* (Å)	7.0242 (6), 6.2316 (6), 17.1574 (15)
β (°)	91.473 (7)
*V* (Å^3^)	750.77 (12)
*Z*	2
Radiation type	Mo *K*α
μ (mm^−1^)	0.11
Crystal size (mm)	0.17 × 0.13 × 0.04

Data collection	
Diffractometer	Stoe *IPDS* II two-circle
Absorption correction	Multi-scan (*X-AREA*; Stoe & Cie, 2001 ▸)
*T*_min_, *T*_max_	0.362, 1.000
No. of measured, independent and observed [*I* > 2σ(*I*)] reflections	7746, 1532, 1285
*R*_int_	0.036
(sin θ/λ)_max_ (Å^−1^)	0.625

Refinement	
*R*[*F* ^2^ > 2σ(*F* ^2^)], *wR*(*F* ^2^), *S*	0.041, 0.101, 1.08
No. of reflections	1532
No. of parameters	109
H-atom treatment	H-atom parameters constrained
Δρ_max_, Δρ_min_ (e Å^−3^)	0.18, −0.16

Computer programs: *X-AREA* (Stoe & Cie, 2001 ▸), *SHELXS97* (Sheldrick, 2008 ▸), *SHELXL2014* (Sheldrick, 2015 ▸) and *XP* in *SHELXTL-Plus* (Sheldrick, 2008 ▸).

## supplementary crystallographic information

### Crystal data

**Table:**

C_18_H_18_F_2_N_2_O_2_	*F*(000) = 348
*M**_r_* = 332.34	*D*_x_ = 1.470 Mg m^−^^3^
Monoclinic, *P*2_1_/*c*	Mo *K*α radiation, λ = 0.71073 Å
*a* = 7.0242 (6) Å	Cell parameters from 7746 reflections
*b* = 6.2316 (6) Å	θ = 3.5–27.8°
*c* = 17.1574 (15) Å	µ = 0.11 mm^−^^1^
β = 91.473 (7)°	*T* = 173 K
*V* = 750.77 (12) Å^3^	Plate, colourless
*Z* = 2	0.17 × 0.13 × 0.04 mm

### Data collection

**Table:**

Stoe IPDS II two-circle diffractometer	1285 reflections with *I* > 2σ(*I*)
Radiation source: Genix 3D IµS microfocus X-ray source	*R*_int_ = 0.036
ω scans	θ_max_ = 26.4°, θ_min_ = 3.5°
Absorption correction: multi-scan (X-AREA; Stoe & Cie, 2001)	*h* = −8→8
*T*_min_ = 0.362, *T*_max_ = 1.000	*k* = −7→7
7746 measured reflections	*l* = −20→21
1532 independent reflections	

### Refinement

**Table:**

Refinement on *F*^2^	0 restraints
Least-squares matrix: full	Hydrogen site location: inferred from neighbouring sites
*R*[*F*^2^ > 2σ(*F*^2^)] = 0.041	H-atom parameters constrained
*wR*(*F*^2^) = 0.101	*w* = 1/[σ^2^(*F*_o_^2^) + (0.0538*P*)^2^ + 0.1464*P*] where *P* = (*F*_o_^2^ + 2*F*_c_^2^)/3
*S* = 1.08	(Δ/σ)_max_ < 0.001
1532 reflections	Δρ_max_ = 0.18 e Å^−^^3^
109 parameters	Δρ_min_ = −0.16 e Å^−^^3^

### Special details

**Table:**

Geometry. All esds (except the esd in the dihedral angle between two l.s. planes) are estimated using the full covariance matrix. The cell esds are taken into account individually in the estimation of esds in distances, angles and torsion angles; correlations between esds in cell parameters are only used when they are defined by crystal symmetry. An approximate (isotropic) treatment of cell esds is used for estimating esds involving l.s. planes.

### Fractional atomic coordinates and isotropic or equivalent isotropic displacement parameters (Å^2^)

**Table:**

	*x*	*y*	*z*	*U*_iso_*/*U*_eq_	
F1	0.80087 (14)	0.60837 (18)	0.86796 (5)	0.0408 (3)	
O1	0.69940 (16)	0.06550 (17)	0.61834 (6)	0.0293 (3)	
N1	0.73058 (17)	0.3605 (2)	0.52754 (7)	0.0256 (3)	
C1	0.5232 (2)	0.3961 (2)	0.52148 (8)	0.0260 (3)	
H1A	0.4706	0.4020	0.5744	0.031*	
H1B	0.4624	0.2743	0.4934	0.031*	
C2	0.7756 (2)	0.1410 (3)	0.54504 (9)	0.0294 (4)	
H2A	0.9158	0.1242	0.5471	0.035*	
H2B	0.7249	0.0493	0.5022	0.035*	
C3	0.72790 (19)	0.2069 (2)	0.67944 (8)	0.0237 (3)	
C4	0.79289 (19)	0.4168 (2)	0.66810 (8)	0.0228 (3)	
C5	0.8283 (2)	0.4965 (3)	0.58624 (8)	0.0267 (3)	
H5A	0.7820	0.6459	0.5809	0.032*	
H5B	0.9668	0.4961	0.5770	0.032*	
C6	0.81932 (19)	0.5507 (3)	0.73252 (8)	0.0250 (3)	
H6	0.8647	0.6931	0.7263	0.030*	
C7	0.7786 (2)	0.4732 (3)	0.80519 (8)	0.0273 (3)	
C8	0.7114 (2)	0.2675 (3)	0.81752 (9)	0.0293 (4)	
H8	0.6837	0.2193	0.8685	0.035*	
C9	0.6854 (2)	0.1337 (3)	0.75374 (9)	0.0269 (3)	
H9	0.6386	−0.0079	0.7606	0.032*	

### Atomic displacement parameters (Å^2^)

**Table:**

	*U*^11^	*U*^22^	*U*^33^	*U*^12^	*U*^13^	*U*^23^
F1	0.0442 (6)	0.0526 (7)	0.0255 (5)	−0.0053 (5)	0.0007 (4)	−0.0122 (4)
O1	0.0393 (6)	0.0227 (6)	0.0258 (6)	−0.0001 (4)	0.0008 (4)	−0.0013 (4)
N1	0.0251 (6)	0.0299 (7)	0.0217 (6)	0.0008 (5)	0.0008 (4)	0.0007 (5)
C1	0.0248 (7)	0.0289 (8)	0.0243 (7)	−0.0015 (6)	0.0006 (5)	0.0018 (6)
C2	0.0338 (8)	0.0310 (8)	0.0233 (7)	0.0046 (6)	0.0025 (6)	−0.0032 (6)
C3	0.0227 (7)	0.0253 (8)	0.0231 (7)	0.0034 (6)	−0.0003 (5)	0.0000 (6)
C4	0.0202 (6)	0.0264 (8)	0.0220 (7)	0.0014 (5)	0.0008 (5)	0.0018 (6)
C5	0.0273 (7)	0.0296 (8)	0.0232 (7)	−0.0033 (6)	0.0000 (5)	0.0032 (6)
C6	0.0210 (6)	0.0269 (8)	0.0271 (7)	−0.0004 (5)	0.0000 (5)	0.0003 (6)
C7	0.0245 (7)	0.0351 (9)	0.0223 (7)	0.0020 (6)	−0.0019 (5)	−0.0057 (6)
C8	0.0262 (7)	0.0399 (9)	0.0218 (7)	0.0026 (6)	0.0036 (6)	0.0059 (7)
C9	0.0252 (7)	0.0269 (8)	0.0287 (8)	0.0014 (6)	0.0024 (6)	0.0060 (6)

### Geometric parameters (Å, º)

**Table:**

F1—C7	1.3731 (17)	C3—C4	1.401 (2)
O1—C3	1.3803 (18)	C4—C6	1.393 (2)
O1—C2	1.4574 (18)	C4—C5	1.5161 (19)
N1—C2	1.434 (2)	C5—H5A	0.9900
N1—C5	1.4721 (19)	C5—H5B	0.9900
N1—C1	1.4746 (18)	C6—C7	1.374 (2)
C1—C1^i^	1.522 (3)	C6—H6	0.9500
C1—H1A	0.9900	C7—C8	1.384 (2)
C1—H1B	0.9900	C8—C9	1.384 (2)
C2—H2A	0.9900	C8—H8	0.9500
C2—H2B	0.9900	C9—H9	0.9500
C3—C9	1.393 (2)		
			
C3—O1—C2	113.57 (12)	C6—C4—C5	121.16 (14)
C2—N1—C5	108.03 (12)	C3—C4—C5	119.75 (13)
C2—N1—C1	111.72 (12)	N1—C5—C4	111.15 (12)
C5—N1—C1	113.84 (12)	N1—C5—H5A	109.4
N1—C1—C1^i^	111.15 (14)	C4—C5—H5A	109.4
N1—C1—H1A	109.4	N1—C5—H5B	109.4
C1^i^—C1—H1A	109.4	C4—C5—H5B	109.4
N1—C1—H1B	109.4	H5A—C5—H5B	108.0
C1^i^—C1—H1B	109.4	C7—C6—C4	118.88 (15)
H1A—C1—H1B	108.0	C7—C6—H6	120.6
N1—C2—O1	113.88 (12)	C4—C6—H6	120.6
N1—C2—H2A	108.8	F1—C7—C6	118.28 (15)
O1—C2—H2A	108.8	F1—C7—C8	118.74 (13)
N1—C2—H2B	108.8	C6—C7—C8	122.95 (14)
O1—C2—H2B	108.8	C7—C8—C9	118.40 (13)
H2A—C2—H2B	107.7	C7—C8—H8	120.8
O1—C3—C9	117.09 (14)	C9—C8—H8	120.8
O1—C3—C4	122.16 (13)	C8—C9—C3	119.93 (15)
C9—C3—C4	120.75 (14)	C8—C9—H9	120.0
C6—C4—C3	119.07 (13)	C3—C9—H9	120.0
			
C2—N1—C1—C1^i^	−161.85 (15)	C1—N1—C5—C4	75.31 (15)
C5—N1—C1—C1^i^	75.45 (18)	C6—C4—C5—N1	−160.16 (12)
C5—N1—C2—O1	65.92 (15)	C3—C4—C5—N1	18.20 (18)
C1—N1—C2—O1	−60.03 (16)	C3—C4—C6—C7	−0.8 (2)
C3—O1—C2—N1	−45.71 (17)	C5—C4—C6—C7	177.62 (13)
C2—O1—C3—C9	−170.60 (12)	C4—C6—C7—F1	−178.47 (12)
C2—O1—C3—C4	10.67 (19)	C4—C6—C7—C8	−0.3 (2)
O1—C3—C4—C6	−179.71 (12)	F1—C7—C8—C9	178.65 (13)
C9—C3—C4—C6	1.6 (2)	C6—C7—C8—C9	0.5 (2)
O1—C3—C4—C5	1.9 (2)	C7—C8—C9—C3	0.4 (2)
C9—C3—C4—C5	−176.79 (13)	O1—C3—C9—C8	179.82 (13)
C2—N1—C5—C4	−49.39 (15)	C4—C3—C9—C8	−1.4 (2)

Symmetry code: (i) −*x*+1, −*y*+1, −*z*+1.

### Hydrogen-bond geometry (Å, º)

*Cg*2 is the centroid of the C3/C4/C6/C7/C8/C9 ring

**Table:**

*D*—H···*A*	*D*—H	H···*A*	*D*···*A*	*D*—H···*A*
C2—H2*A*···F1^ii^	0.99	2.44	3.300 (2)	145
C6—H6···*Cg*2^iii^	0.95	2.88	3.527 (2)	126
C9—H9···*Cg*2^iv^	0.95	2.90	3.577 (2)	129

Symmetry codes: (ii) −*x*+2, *y*−1/2, −*z*+3/2; (iii) −*x*+2, *y*+1/2, −*z*+3/2; (iv) −*x*+1, *y*−1/2, −*z*+3/2.
